# Keratinocyte Priming by *Staphylococcus aureus* Reduces HSV‐1 Susceptibility

**DOI:** 10.1111/all.70392

**Published:** 2026-05-20

**Authors:** Phila Cara Baumann, Rebecca Pospich, Katinka Döhner, Ilona Klug, Julio Cesar Villalvazo Guerrero, Jan Hartmann, Inken Harder, Wolfgang Lieb, Barbara Kind, Andreas Kleinheinz, Susanne Abraham, Matthias Augustin, Elke Weisshaar, Jochen Schmitt, Stephan Weidinger, Beate Sodeik, Thomas Werfel, Lennart M. Roesner, Stephan Traidl, The TREATgermany Study Group

**Affiliations:** ^1^ Department of Dermatology and Allergy Hannover Medical School Hannover Germany; ^2^ Institute of Virology Hannover Medical School Hannover Germany; ^3^ German Centre for Infection Research (DZIF) Hannover‐Braunschweig Site Hannover Germany; ^4^ Department of Dermatology and Allergy University Hospital Schleswig‐Holstein Kiel Germany; ^5^ Institute of Epidemiology Kiel University Kiel Germany; ^6^ Center for Evidence‐Based Health Care (ZEGV), Faculty of Medicine Carl Gustav Carus TU Dresden Dresden Germany; ^7^ Clinics for Dermatology Elbe Klinikum Buxtehude Buxtehude Germany; ^8^ Department of Dermatology, Faculty of Medicine Carl Gustav Carus, University Allergy Center TU Dresden Dresden Germany; ^9^ Institute for Health Services Research in Dermatology Hamburg University Medical Center Hamburg Eppendorf Hamburg Germany; ^10^ Occupational Dermatology, Department of Dermatology Ruprecht‐Karls University Heidelberg Heidelberg Germany; ^11^ Cluster of Excellence RESIST (EXC 2155) Hannover Medical School Hannover Germany

**Keywords:** atopic dermatitis, eczema herpeticum, herpes simplex virus, skin microbiota, *Staphylococcus* spp, Th2 cytokines

## Abstract

**Background:**

Individuals with atopic dermatitis (AD) are at increased risk for skin infections, including eczema herpeticum (EH), a severe condition caused by herpes simplex virus type 1 (HSV‐1). While AD skin is often colonized by *Staphylococcus aureus* ( *S. aureus* ), its role in EH susceptibility remains unclear. Here we aim to investigate differences in the skin microbiome of AD patients with (ADEH^+^) and without (ADEH^−^) EH and examine the impact of *S. aureus* and *S. epidermidis* on HSV‐1 infection in an in vitro keratinocyte model.

**Methods:**

16S microbiome sequencing was performed on skin samples from ADEH^+^, ADEH^−^, and healthy controls. To investigate microbial effects on HSV‐1 infection, keratinocytes were pre‐incubated with heat‐killed *S. aureus* (HKSA) or *S. epidermidis* (HKSE), followed by HSV‐1 infection in the presence or absence of the Th2 cytokines IL‐4 and IL‐13, simulating the conditions of AD lesional skin. Infection rates and transcriptomic changes were analyzed.

**Results:**

ADEH^+^ patients showed a reduced microbial diversity compared to ADEH^−^, with increased *S. aureus* and *S. epidermidis* colonization. HKSA, but not HKSE, protected keratinocytes from HSV‐1 infection and reduced the release of infectious progeny virus. Transcriptome analysis of keratinocytes revealed HKSA‐induced upregulation of interferon pathways and antimicrobial peptides, and downregulation of HSV‐1 entry factors.

**Conclusion:**

Pre‐incubation with *S. aureus* set basal keratinocytes into an alarmed state, restricting HSV‐1 infection presumably via downregulation of receptors important for viral entry and activation of antiviral pathways.

## Graphical abstract

ADEH^+^ patients have a reduced microbial diversity with increased *S. aureus* and *S. epidermidis* colonization compared to ADEH^−^ and healthy controls. Heat‐killed *S. aureus* reduced HSV‐1 susceptibility and release of infectious progeny virus in a keratinocyte in vitro model. Heat‐killed *S. aureus* induced IFN pathway and AMP upregulation and downregulation of HSV‐1 entry factors, priming keratinocytes into an antiviral state. ADEH ^−^, atopic dermatitis patients without a history of eczema herpeticum; ADEH ^+^, atopic dermatitis patients with a history of eczema herpeticum; AMPs, antimicrobial peptides; GFP, green fluorescent protein; HSV‐1, herpes simplex virus 1; IFN, interferon; MFI, mean fluorescence intensity; RNAseq, RNA sequencing; RT‐qPCR, quantitative reverse transcription polymerase chain reaction; *S.aureus*, *Staphylococcus aureus* ; *S.epidermidis*, *Staphylococcus epidermidis;* TREATgermany, German Clinical Registry: Therapy and medical care for patients with moderate to severe atopic dermatitis.

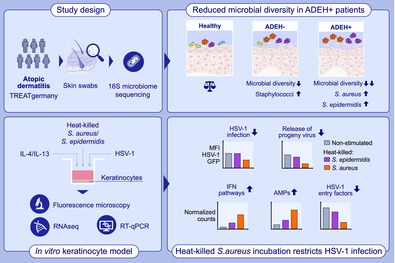

## 1 Introduction

Atopic dermatitis (AD) is a common inflammatory skin condition characterized by intense pruritus and a multifactorial pathogenesis [1]. The disease is associated with an increased susceptibility to bacterial, viral, and fungal skin infections [2]. Eczema herpeticum (EH) is a potential life‐threatening and often recurrent viral infection caused by herpes simplex virus type 1 (HSV‐1), and probably less frequently by HSV‐2, that occurs in a subset of AD patients on eczematous skin [3, 4].

The human skin microbiota is essential for skin physiology and immune function. These microorganisms play a role in the skin's barrier function by protecting against invading pathogens and also contribute to the development of the skin's innate and adaptive immune system [5]. Staphylococci are typical microbes found on healthy skin, including the commensal *Staphylococcus epidermidis* ( *S. epidermidis* ), which plays a role in maintaining the barrier functions by protecting the host from pathogenic bacteria [6]. The majority of the skin microbiota is commensal or mutualistic under homeostatic conditions but can be biased towards a more pathogenic behavior. A disrupted barrier and an imbalance between host and microorganisms and between commensal and pathogenic bacteria can result in the development of cutaneous and sometimes also subsequent systemic infections [7].

Several studies demonstrated that the skin microbiota diversity in AD is changed towards a generally decreased bacterial diversity along with a strong colonization with *Staphylococcus aureus* ( *S. aureus* ), especially in the lesional areas [8]. *S. aureus* influences the disease in multiple ways by inducing barrier disruption and inflammation, and producing virulence factors that directly induce itch [9, 10]. However, it remains unclear whether the microbiome of AD patients with a history of EH (ADEH^+^) is altered at all compared to patients without a history of EH (ADEH^−^), and whether the most abundant species on the skin influence HSV‐1 virus infection.

In the present study, we developed an in vitro model to investigate the impact of the most common species on AD skin, *S. epidermidis* and *S. aureus* , on HSV‐1 infection of keratinocytes. We used keratinocytes as they represent the majority of epidermal cells and are a source and target of various cytokines that affect the immune responses of the skin. As AD is mainly a Th2 driven disease, we used the Th2 cytokines interleukin (IL)‐4 and IL‐13 to mimic the cytokine milieu of AD lesional skin. Heat‐killed *S. epidermidis* and *S. aureus* were added to mimic skin colonization rather than skin infection, followed by subsequent HSV‐1 infection to investigate how a previous challenge with staphylococci influences keratinocyte gene expression and subsequent HSV‐1 infection.

Our findings indicate that heat‐killed *S. aureus* , but not *S. epidermidis* , provides protection against HSV‐1 infection.

## 2 Methods

### 2.1 Ethics Statement

Samples were collected after informed consent was given within the TREATgermany registry, which has been approved by the responsible ethics committees (No. EK TUD 118032016, EK CAU AZ: B261/16) and is listed at the clinicaltrials.gov database (NCT03057860). A comprehensive overview of the inclusion criteria, methods, and objectives of the TREATgermany registry has been previously published [11, 12, 13]. Briefly, adult patients aged ≥ 18 years with a diagnosis of moderate to severe AD, based on the UK Working Party diagnostic criteria (objective Scoring Atopic Dermatitis (oSCORAD) > 20, assessed by trained physicians or board‐certified dermatologists, and/or current or prior systemic therapy for AD within the last 24 months), were enrolled from routine dermatological care settings, including both university and non‐university hospitals, as well as dermatology practices. Methodological Details are provided in the Supporting Information Methods.

### 2.2 16S Microbiome Analysis

Detailed methods regarding the swabs collected in the TREATgermany registry were described previously [14]. Shortly, patients were asked to avoid bathing, showering, or applying topical agents for 24 h before the sampling visit. For the present analysis, the results from the swabs taken on the antecubital fossa were used. Statistical analyses were performed using R (version 4.2.3) [15] with an RStudio IDE (version 2023.03) [16] within a macOS Monterey environment. Microbiome data were analyzed using the phyloseq (version 1.46.0) [17], microbiome (version 1.24.0) [18], and vegan packages (version 2.6‐8) [4]. The Shannon index was calculated based on non‐rarefied data.

### 2.3 Keratinocyte Cell Culture

Human primary keratinocytes were prepared from foreskin as described before [19]. Cells were cultured in keratinocyte growth medium 2 (Keratinocyte Basal Medium 2 plus supplements, PromoCell) without antibiotics at 37°C in a 5% CO_2_ atmosphere. Cells were passaged at around 80% confluence. Cells were then either split for further culture or plated for experiments.

### 2.4 Vero Cell Culture

Vero CCL‐81 cells were cultured in MEM Eagle medium with Earle's Balanced Salt Solution and L‐glutamine (CytoGen GmbH) containing 7.5% FBS (Capricorn Scientific GmbH) and 1% Non‐Essential Amino Acids (CytoGen GmbH) and incubated at 37°C in a 5% CO_2_ atmosphere. Cells were passaged at ~80%–90% confluence as described in the supplements for keratinocytes, except that the trypsin inhibitor was omitted and Vero culture media was added instead.

### 2.5 Bacterial Culture and Heat‐Killing

*S. aureus* (NCTC 8325) and *S. epidermidis* (DSM 20044, ATCC 14990) were grown on Columbia blood agar plates (5% sheep blood, Becton Dickinson) at 37°C. Subcolonies were transferred to tryptic soy broth (TSB; Sigma‐Aldrich) and incubated overnight at 37°C with shaking. Cultures were diluted (1:10) in TSB, grown for 3 h, centrifuged (5000 *g*, 5 min, 20°C), washed with DPBS, and resuspended in keratinocyte basal medium (0.06 mM CaCl_2_, 100 μg/mL gentamicin). Bacterial concentration was adjusted to the appropriate optical density (OD). An OD of 0.2 was used for HKSE and HKSA unless otherwise specified. Heat‐killing was performed at 65°C for 1 h in a water bath.

### 2.6 Keratinocyte In Vitro Model

Keratinocytes were seeded (10^4^ cells/well) in 96‐well plates and incubated overnight in growth medium. Cells were stimulated with IL‐4 and IL‐13 (40 ng/mL each) for 24 h or left untreated, then exposed to HKSA/HKSE in basal medium overnight at 37°C, 5% CO_2_.

### 2.7 HSV‐1 Infection of Keratinocytes

Keratinocytes were precooled with CO_2_‐independent medium (0.1% BSA) and infected with HSV‐1 (17+) Lox‐pMCMV‐GFP (10^6^ PFU/mL) for 2 h on ice. After washing, basal medium was added, and cells were incubated for 20 h at 37°C. Supernatants were collected for Vero cell infection, and keratinocytes were fixed with 4% formaldehyde and stained with DAPI and incubated for 20 min at room temperature. Cells were washed with DPBS and overlayed with ddH_2_O. The plates were stored in the dark at 4°C until measurement of DAPI and GFP with an automated microscope (Cytation 3, BioTek Instruments Inc.).

### 2.8 Vero Cell Infection

The supernatants collected from keratinocytes after 20 h of HSV‐1 infection were used to infect indicator Vero cells to compare the amount of infectious HSV‐1 released from the differentially treated keratinocytes. Vero cells (6000 cells/well in 50 μL Vero cell medium) were plated into 96‐well plates and incubated overnight at 37°C. The medium was discarded and 50 μL of HSV‐1 keratinocyte supernatant (1:2 in Vero cell medium) was added. After 20 h incubation at 37°C, the supernatant was removed and the Vero cells were fixed and stained as described above for further analysis.

### 2.9 Microscopy and Image Analysis

Fixed keratinocytes and Vero cells were imaged using a Cytation 3 reader (BioTek Instruments Inc.) with DAPI and GFP channels. Four central images per well were analyzed using CellProfiler software to quantify GFP intensity.

### 2.10 Cell Viability Assay

Cell viability was assessed with the RealTime‐Glo MT Cell Viability Assay (Promega) following exposure to HKSE/HKSA. Luminescence was measured after 1 h incubation at 37°C using a FluoStar Optima reader.

### 2.11 Bulk RNA Sequencing

RNA was extracted using the ReliaPrep RNA Cell Miniprep System (Promega). cDNA was synthesized with the RevertAid Kit (Thermo Scientific), and RT‐qPCR was performed for various targets. Libraries were prepared, sequenced, and analyzed using the DESeq2 pipeline. Genes with LFC > 2 and FDR < 0.05 were considered differentially expressed.

### 2.12 Statistical Analysis

For statistical analysis the software Graph Pad Prism 8.4.3 (GraphPad Software Inc) was used. Data were analyzed for normal distribution using the Shapiro–Wilk‐test. Normally distributed data were analyzed with Repeated Measures ANOVA with Dunnett's multiple comparison test and data with a non‐Gaussian distribution were analyzed with Friedman test for paired data and uncorrected Dunn's multiple comparison test or Kruskal‐Wallis test for unpaired data. *p* values ≤ 0.05 were considered as significant. *p < 0.05, **p < 0.01, ***p < 0.001, ****p < 0.0001.

## 3 Results

### 3.1 AD Patients With a History of Eczema Herpeticum (ADEH ^+^) Showed Reduced Skin Microbiota Diversity

**FIGURE 1:**
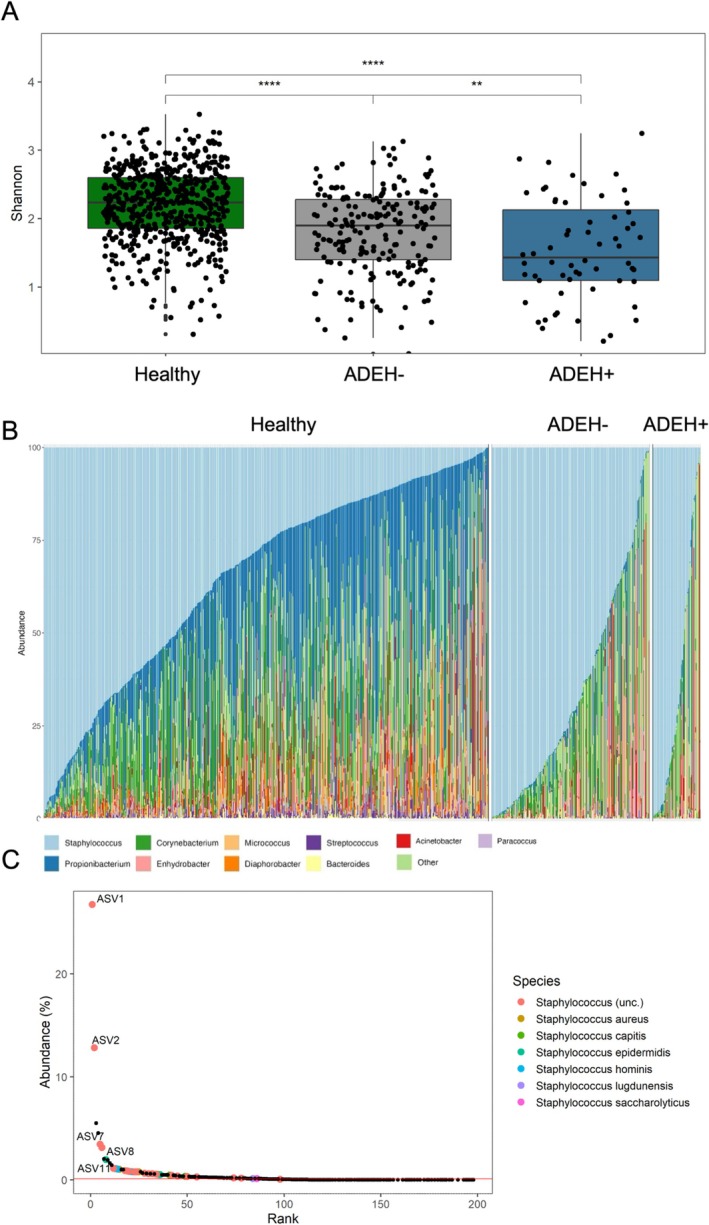
16S Microbiome analysis of swabs from the cubital fossa of healthy individuals and AD patients without (ADEH^−^) and with (ADEH^+^) a history of eczema herpeticum (EH). (A) Alpha diversity measured by Shannon index of healthy individuals, ADEH^−^ and ADEH^+^ patients. (B) Relative abundance of different genera. (C) Relative abundance of the individual amplicon sequence variants (ASV) and the corresponding species. Staphylococcus (unc.) denotes ASVs that could not be mapped to a specific *Staphylococcus* species.

Patients with AD have an altered skin microbiota with a dominance of *S. aureus* [20]. However, the skin microbiota in AD patients with a history of eczema herpeticum (ADEH^+^) has not been studied. Therefore, a total of 293 participants, 221 ADEH^−^ patients and 72 ADEH^+^ patients (Figure S1), were included in this study. There were no significant differences between the two groups in terms of age (ADEH^+^ [mean ± SD]: 42.6 ± 14.3 vs. ADEH^−^: 40.4 ± 15.2), gender distribution (females: ADEH^+^ 48.6% vs. ADEH^−^ 43.0%), or disease severity as measured by the Eczema Area and Severity Index (EASI) (ADEH^+^ [mean ± SD]: 17.9 ± 12.3 vs. ADEH^−^: 17.4 ± 13.0). Additionally, a group of 258 healthy controls (age [mean ± SD]: 65.0 ± 11.0, female: 42.0%) were added. Skin swabs were taken and the 16S rRNA genes were amplified and sequenced. Shannon diversity index was used to assess the microbial diversity (Figure 1A). The healthy group exhibited a significantly higher microbial diversity (median Shannon index = 2.2) compared to the ADEH^−^ (1.9) and ADEH^+^ (1.4) groups. Of note, the ADEH^+^ group had significantly lower diversity than the ADEH^−^ group (**p < 0.01). Investigating richness and evenness showed significant differences in both measurements (data not shown). ADEH^−^ patients' skin microbiota was dominated by *Staphylococcus* (light blue), *Corynebacterium* (green), and *Propionibacterium* (dark blue) species (Figure 1B). In the ADEH^+^ group, staphylococci dominance was even more pronounced, indicating a reduction in microbial diversity and a potential overgrowth of specific taxa. Analyzing the abundance of the different amplicon sequence variants (ASVs) revealed that the most abundant ASVs in AD belong to staphylococci (Figure 1C). In particular, *Staphylococcus* ASV1 and ASV2 were highly abundant in ADEH^+^ patients, while *S. aureus* , *S. epidermidis* , *Staphylococcus hominis* ( *S. hominis* ), and *Staphylococcus capitis* ( *S. capitis* ) were among the most prevalent species. Applying the BLAST analysis manually to the ASV1 and ASV2 revealed that they were most likely *S. aureus* and *S. epidermidis* , respectively. These findings suggest that different staphylococci, especially *S. aureus* and *S. epidermidis* , may influence the viral susceptibility.

### 3.2 *S. aureus* but Not *S. epidermidis* Reduced HSV‐1 Infection in Keratinocytes In Vitro

**FIGURE 2:**
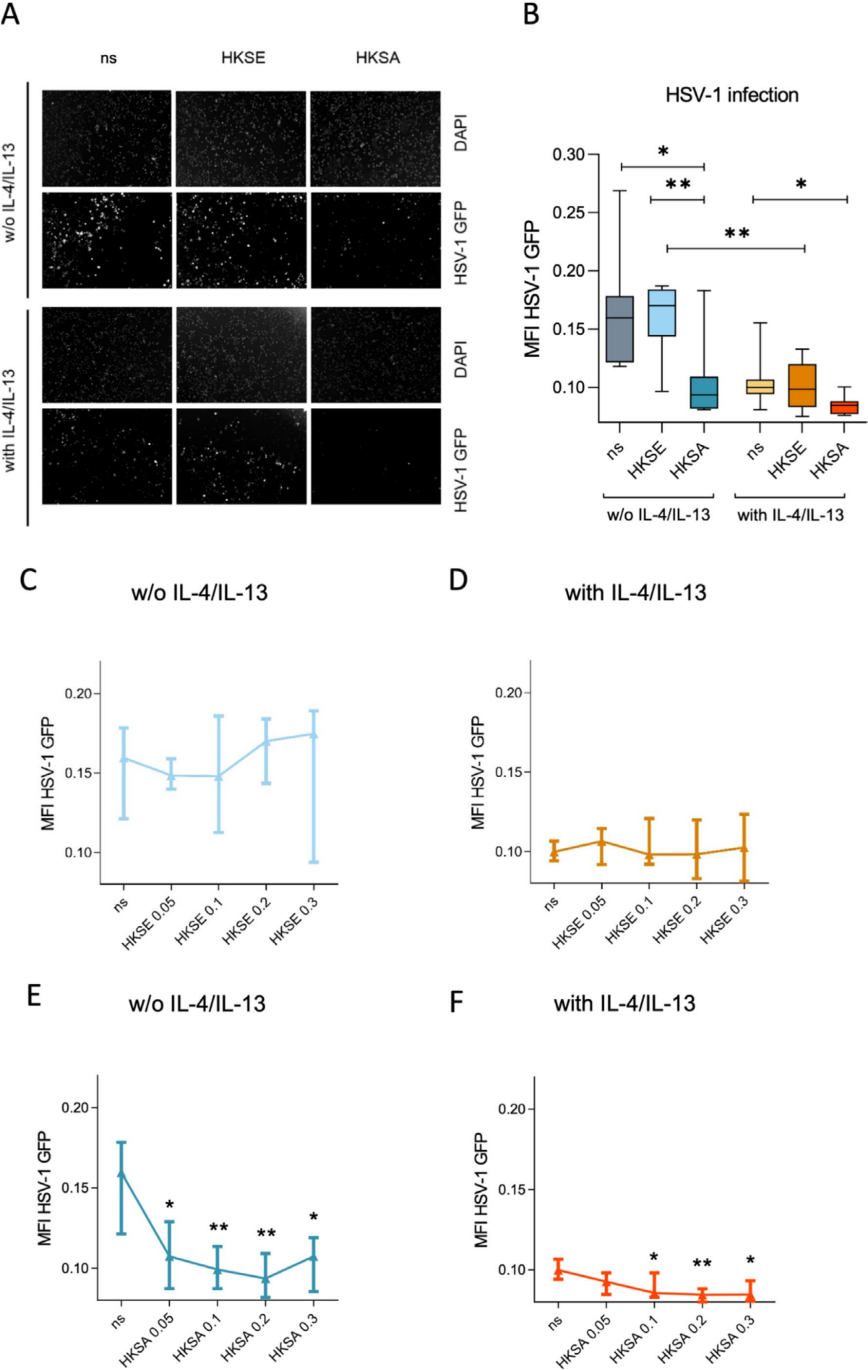
Infection of NHEKs with HSV‐1 after stimulation with heat‐killed staphylococci. (A) Representative fluorescence images of NHEKs infected with 1 × 10^6^ PFU/mL HSV1‐GFP after stimulation with heat‐killed *S. epidermidis* (HKSE; OD 0.2) or *S. aureus* (HKSA; OD 0.2) in the presence or absence of IL‐4 and IL‐13 (40 ng/mL). Nuclei are stained with DAPI. (B) Mean fluorescence intensity of HSV1‐GFP (*n* = 7 independent NHEK donors). Box‐and‐whisker plots range from minimum to maximum values, and lower and upper quartiles, as well as medians are shown. Mean fluorescence intensity of background 0.079. (C–F) Influence of staphylococci in different concentrations (OD 0.05–0.3) on HSV‐1 infection. Median and interquartile range are shown. For all, statistical significance calculated by Friedman test with Dunn‘s multiple comparison test, * < 0.05, ** < 0.01, *** < 0.001.

To investigate the influence of the two most abundant bacterial species, *S. aureus* and *S. epidermidis* , on ADEH^+^ skin and subsequent HSV‐1 infection, we established an in vitro keratinocyte model. Since EH occurs mostly by reactivation of HSV‐1 and transfer of progeny virus from nerve endings to basal keratinocytes [21], we employed undifferentiated Normal Human Epidermal Keratinocytes (NHEKs). To model skin colonization, we used heat‐killed bacteria that were not able to overgrow NHEK culture and did not affect the viability of NHEKs by producing toxins, such as α‐toxin. To evaluate if *S. aureus* or *S. epidermidis* can influence HSV‐1 viral susceptibility in AD, we treated keratinocytes with heat‐killed *S. epidermidis* (HKSE) and *S. aureus* (HKSA) at different concentrations, with or without IL‐4/IL‐13 (40 ng/mL) to create an inflammatory AD skin environment. The treatment with the heat‐killed bacteria and cytokines had no effect on cell viability (Figure S2). Pre‐incubation of keratinocytes with HKSA protected keratinocytes from subsequent HSV‐1 infection with the reporter strain HSV1 (17^+^) Lox‐pMCMV‐GFP (HSV1‐GFP) as reflected by a reduced mean GFP fluorescence intensity (Figure 2A,B). Conversely, HKSE had no effect in both conditions, with and without IL‐4/IL‐13 (Figure 2A,B). The Th2 cytokines IL‐4 and IL‐13 reduced HSV‐1 infection (Figure 2B). The reducing effect of HKSA on HSV‐1 infection was present in different concentrations, while HKSE had no effect at any concentration (Figure 2C–F).

### 3.3 Reduced HSV‐1 Replication in Keratinocytes Treated With *S. aureus*

**FIGURE 3:**
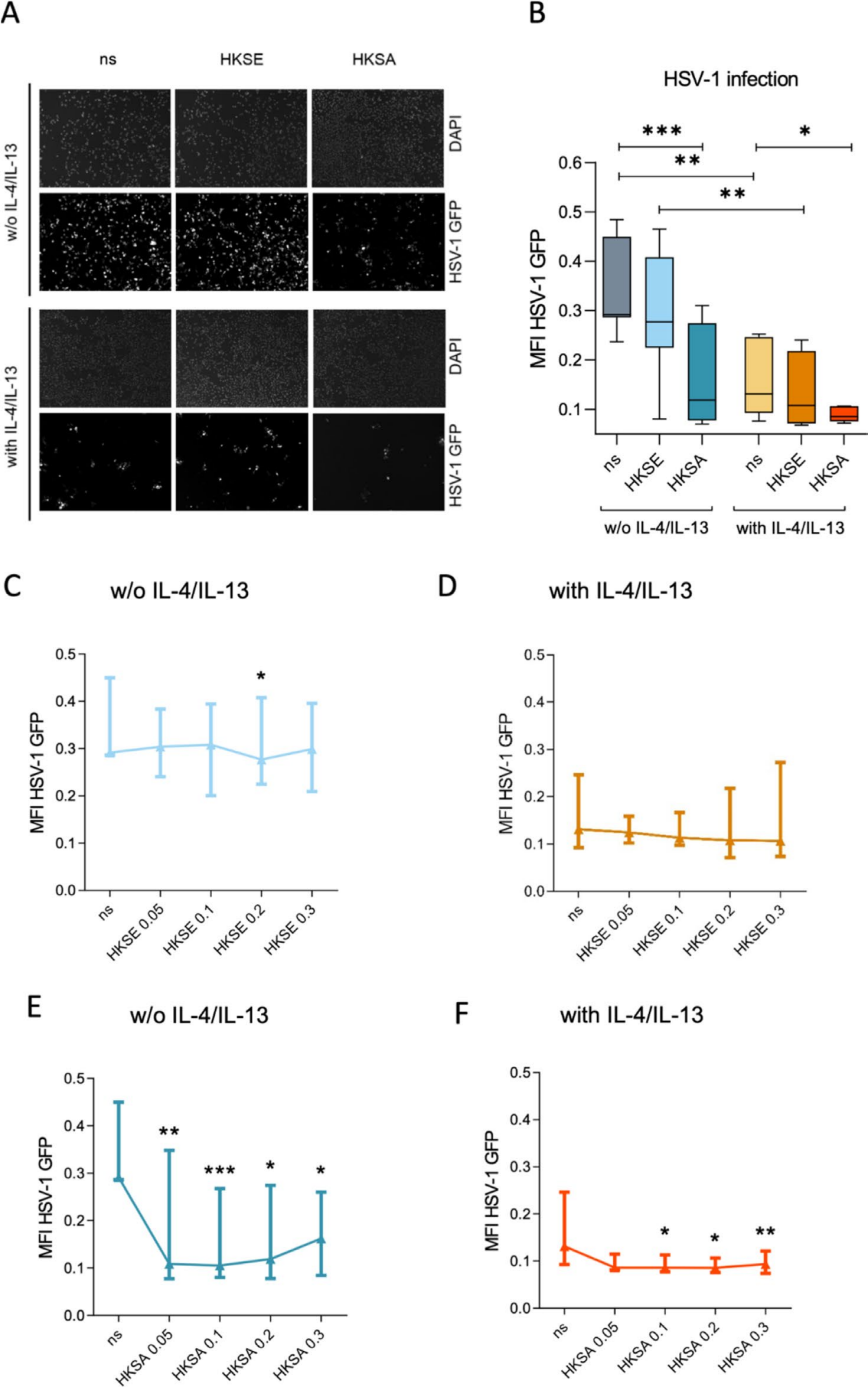
Infection of Vero cells with supernatants of HSV‐1 infected NHEKs after stimulation with heat‐killed staphylococci. (A) Representative fluorescence images of Vero cells infected with HSV1‐GFP (1 × 10^6^ PFU/mL) derived from supernatants of NHEKs that were either non‐stimulated (ns) or stimulated with heat‐killed *S. epidermidis* (HKSE; OD 0.2) or *S. aureus* (HKSA; OD 0.2) in the presence or absence of IL‐4 and IL‐13 (40 ng/mL). Nuclei are stained with DAPI. (B) Mean fluorescence intensity of HSV1‐GFP (*n* = 7 independent experiments). Box‐and‐whisker plots range from minimum to maximum values, and lower and upper quartiles, as well as medians are shown. Mean fluorescence intensity of background 0.076. (C–F) Influence of staphylococci in different concentrations (OD 0.05–0.3) on HSV‐1 infection. Median and interquartile range are shown. For all, statistical significance calculated by Friedman test with Dunn‘s multiple comparison test, * < 0.05, ** < 0.01, *** < 0.001.

To determine the release efficiency of infectious HSV‐1 progeny virus by NHEKs after infection with HSV1‐GFP for 20 h, supernatants were collected and used for the infection of indicator Vero cells. Vero cells showed reduced infection rates with supernatants from keratinocytes that were infected with HSV1‐GFP after HKSA challenge (Figure 3A,B,E,F), while HKSE had no effect (Figure 3A–D). IL‐4 and IL‐13 treatment of keratinocytes also decreased mean GFP intensities in indicator Vero cells, indicating that Th2 cytokines reduced release of infectious HSV‐1 progeny from NHEKs (Figure 3B).

### 3.4 HKSA Induced Gene Expression Changes in Keratinocytes

**FIGURE 4:**
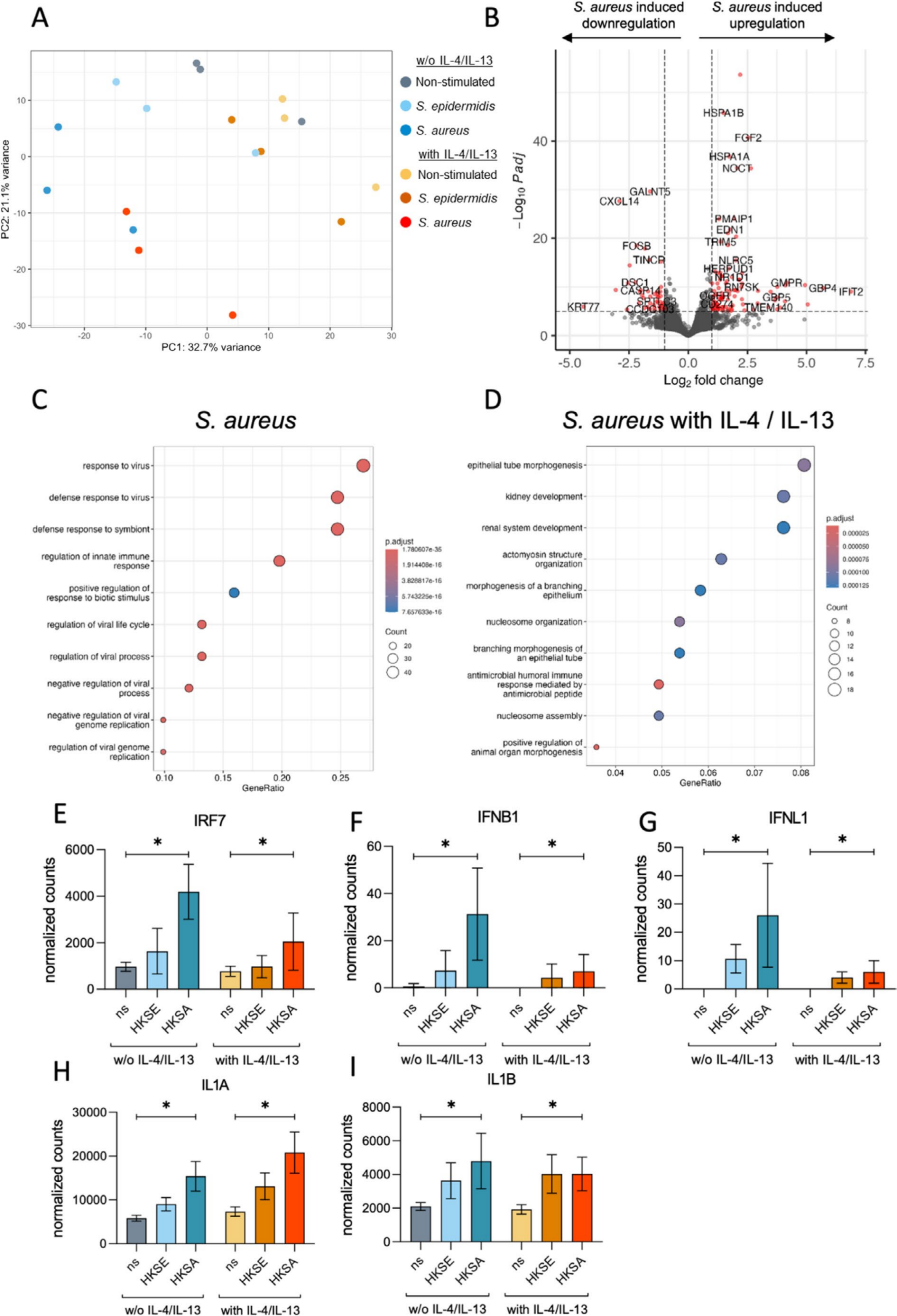
Transcriptomics analysis of keratinocytes stimulated by heat‐killed *S. aureus* (HKSA) and *S. epidermidis* (HKSE). (A) PCA analysis performed with the top *n* = 2000 genes selected by variance. (B) Volcano plot investigated differential expressed genes comparing HKSA stimulated keratinocytes to non‐stimulated keratinocytes in the absence of IL‐4/IL‐13. (C) Gene Ontology Biological Processes pathway analysis of HKSA stimulated keratinocytes and (D) of HKSA stimulated keratinocytes with IL‐4/IL‐13. (E) IRF7, (F) IFNB1 (G) IFNL1, (H) IL1A and (I) IL1B normalized counts in keratinocytes stimulated by HKSE, HKSA or non‐stimulated (ns) with or without IL‐4/IL‐13 (*n* = 3 independent NHEK donors/experiments). Mean with SD is shown, Friedman test with Dunn‘s multiple comparison test was performed for statistical analysis. * < 0.05.

To investigate changes in gene expression, we performed bulk RNA sequencing on HKSA‐ and HKSE‐treated keratinocytes in the presence or absence of IL‐4 and IL‐13 of three independent keratinocyte donors. Principal component analysis (PCA) revealed that keratinocytes incubated with HKSA regardless of cytokine stimulation clustered away from the non‐stimulated and HKSE‐treated keratinocytes (Figure 4A). Analyzing the differentially expressed genes (DEGs, p‐adj. < 0.05, Log_2_FC > |1|), we found 403 DEGs of which 174 were downregulated and 229 were upregulated following HKSA treatment when compared to non‐stimulated keratinocytes (Figure 4B). By contrast, comparison on HKSE‐treated versus non‐stimulated keratinocytes reveals only a limited number of differentially expressed genes (Figure S3A) and to a much lesser extend compared to HKSA‐treated keratinocytes (Figure S3B). Pathway analysis revealed that *S. aureus* induces gene expression involved in virus response, antiviral pathways and pathways involved in innate immune responses (Figure 4C). In the presence of Th2 cytokines, *S. aureus* induced antimicrobial immune responses mediated by antimicrobial peptides (Figure 4D). In addition, the expression of the innate immune response genes IRF7, IFNB1, IFNL1, IL1A, and IL1B was upregulated (Figure 4E–I). The upregulation was validated with additional keratinocyte donors by RT‐qPCR experiments (Figure S4). Furthermore, transcriptomics analysis revealed the upregulation of interferon‐stimulated genes (ISGs) ISG15, MX2, EIF2AK2 and IFIT2 (Figure S5). An analysis of the KEGG pathway “Herpes simplex virus 1 infection” revealed profound upregulation of numerous genes associated with the viral defense response (Figures S6 and S7). Notably, investigating the effects of cytokine stimulation revealed that IL‐4/IL‐13‐stimulated keratinocytes in the absence of *S. aureus* or *S. epidermidis* exhibited no differences in the expression of IRF7, IFNB1, IFNL1, IL1A, and IL1B, or other antiviral immune mediators when compared to non‐stimulated keratinocytes. Investigation of protein‐level effects, assessed by measuring cytokine production in supernatants of NHEKs stimulated with heat‐killed staphylococci in the presence or absence of IL‐4/IL‐13, revealed increased IL‐6 production in keratinocytes stimulated with both *S. aureus* and *S. epidermidis* (Figure S8A). In contrast, IL‐8 upregulation was more pronounced in keratinocytes co‐stimulated with IL‐4/IL‐13 (Figure S8B). Moreover, IL‐1α and IL‐1β secretion was increased after HKSA treatment of keratinocytes (Figure S8C,D).

### 3.5 HKSA Reduced the Expression of Genes Important for HSV‐1 Entry

**FIGURE 5:**
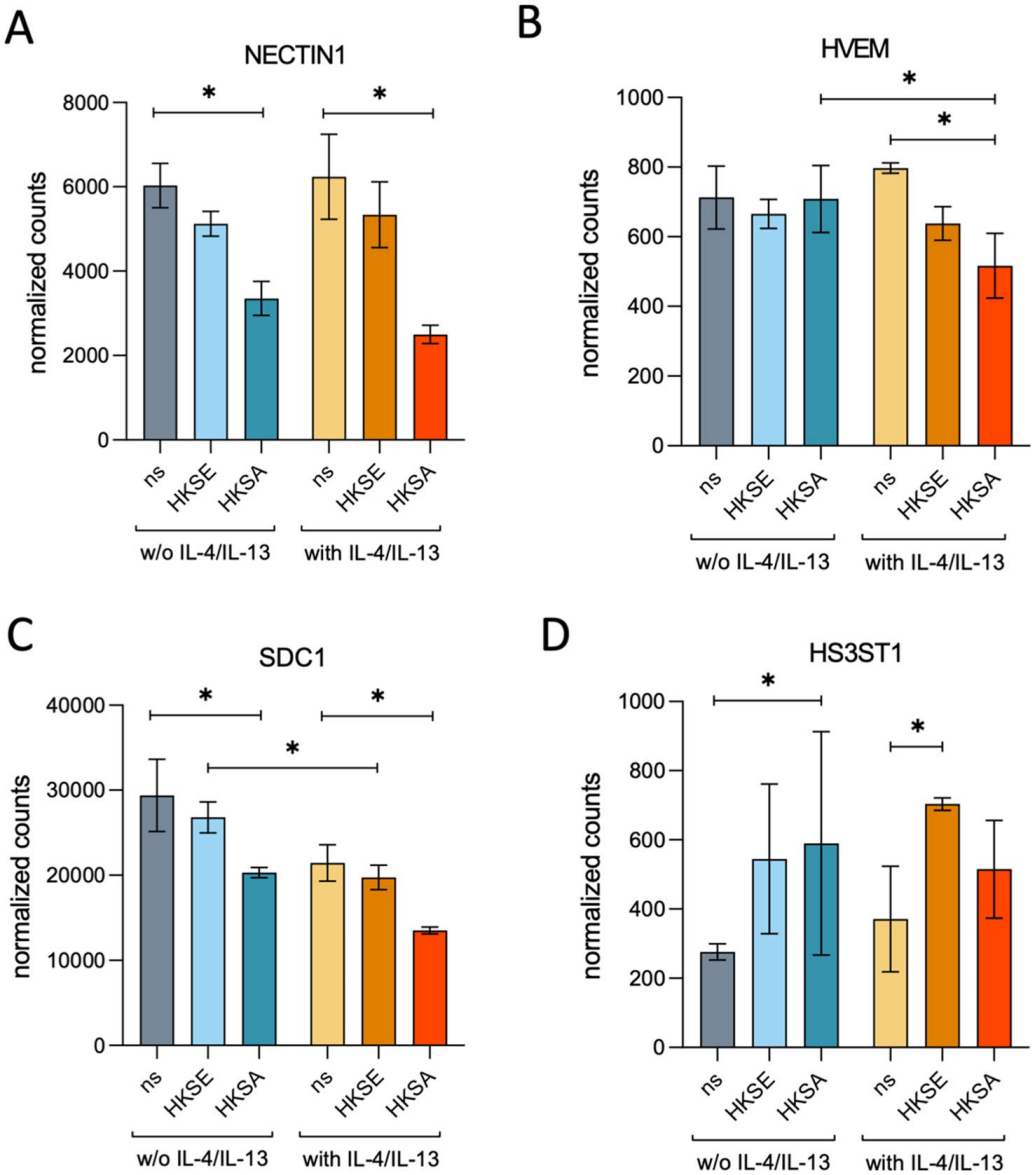
Normalized counts of different expressed genes involved in HSV‐1 cell entry. Gene expression of (A) NECTIN1 (B) HVEM (C) SDC1 (D) HS3ST1 of NHEKs stimulated with heat‐killed *S. aureus* (HKSA) or *S. epidermidis* (HKSE) or non‐stimulated (ns) in combination with or without IL‐4/IL‐13 (*n* = 3 independent experiments/NHEK donors). Mean with SD is shown. Friedman test with Dunn's multiple comparison test was performed for statistical analysis. * < 0.05.

Next, we investigated the effect on genes involved in HSV‐1 entry, replication, and further steps of the infection cycle. HKSA stimulation reduced nectin‐1 expression independently of IL‐4/IL‐13 treatment (Figure 5A and Figure S9A). Nectin‐1 binds the HSV‐1 glycoprotein D (gD) and is the primary receptor mediating HSV‐1 entry into keratinocytes [22, 23, 24]. HVEM is another gene involved in HSV‐1 entry. The combination of IL‐4/IL‐13 and HKSA decreased HVEM expression (Figure 5B and Figure S9B), while no changes were detectable in the absence of cytokines. Syndecan‐1 (SDC1) is a transmembrane heparan sulfate proteoglycan involved in HSV‐1 induced cell‐to‐cell fusion, in viral cell‐to‐cell spread, and in the release of viral particles in the lytic infection cycle [25, 26]. Incubation with HKSA further reduced the expression of SDC1 (Figure 5C and Figure S9C), which may result in a reduced cell‐to‐cell spread. Heparan Sulfate‐Glucosamine 3‐Sulfotransferase 1 gene (HS3ST1) is a gene encoding for a transferase that utilizes 3′‐phospho‐5′‐adenylyl sulfate (PAPS) to catalyze the transfer of a sulfo group to position 3 of glucosamine residues in heparin [27, 28, 29]. The 3‐O‐sulfation is important for the interaction of 3‐O‐sulfated heparin sulfate with HSV‐1 glycoprotein D, which mediates viral entry into cells [30, 31, 32]. Furthermore, an increased expression of HS3ST1 in the presence of bacteria was observed (Figure 5D).

### 3.6 HKSA Induced the Expression of Antimicrobial Peptides Involved in the Defense of HSV‐1

**FIGURE 6:**
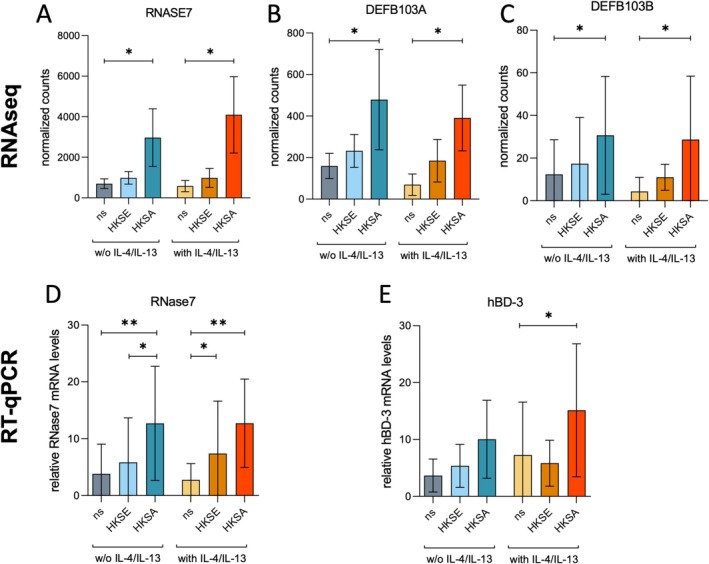
Normalized counts of different expressed genes encoding for antimicrobial peptides. Gene expression of (A) RNASE7, (B) DEFB103A and (C) DEFB103B of keratinocytes stimulated with heat‐killed *S. aureus* (HKSA) or *S. epidermidis* (HKSE) or non‐stimulated (ns) in combination with or without IL‐4/IL‐13 (*n* = 3 independent experiments/NHEK donors). Relative mRNA expression of (D) RNase 7 (*n* = 7) and (E) hBD‐3 (*n* = 5) of NHEKs stimulated with HKSE or HKSA with or without IL‐4/IL‐13 analyzed by RT‐qPCR. Mean with SD is shown. Statistical significance was calculated by Friedman test with Dunn‘s multiple comparison test, * < 0.05, ** < 0.01, *** < 0.001.

Antimicrobial peptides (AMPs) can be induced by bacteria and play an important role in restricting HSV‐1 infection [33, 34] Therefore, we hypothesized that HKSA‐induced AMP induction may contribute to the protective effect on subsequent HSV‐1 infections. Here, we found that HKSA upregulated the expression of RNASE7, DEFB103A and DEFB103B, which encode RNase 7 and human β‐defensin 3 (hBD‐3), respectively (Figure 6A–C). Additionally, mRNA expression of RNase 7 and hBD‐3 was analyzed by RT‐qPCR. Significantly elevated relative RNase 7 mRNA levels in HKSA‐treated keratinocytes and significantly elevated relative hBD‐3 mRNA levels in HKSA‐treated keratinocytes co‐stimulated with IL‐4/IL‐13 confirm these findings of upregulated AMP expression by HKSA (Figure 6D,E).

## 4 Discussion

This is the first study to describe a reduced skin microbiota diversity in ADEH^+^ compared to ADEH^−^ patients, with staphylococcal species, including *S. aureus* and *S. epidermidis* , being the most prevalent. While indicating this strong expansion of staphylococcal ASVs in ADEH^+^ patients, the exact species‐level composition and precise relative abundance of *S. aureus* cannot be conclusively determined due to the limited taxonomic resolution of 16S rRNA sequencing within the genus *Staphylococcus*. The established in vitro model demonstrated that *S. aureus* and Th2 cytokines reduced HSV‐1 infection of keratinocytes, whereas *S. epidermidis* did not influence HSV‐1 infection.

Since EH is mostly caused by reinfection of basal epidermal keratinocytes by HSV‐1 released from nerve endings at the dermal‐epidermal junction upon reactivation, we used undifferentiated keratinocytes for our in vitro model [3]. To mimic the Th2 inflammation, keratinocytes were stimulated with IL‐4 and IL‐13.

Three different receptors are involved in IL‐4 and IL‐13 signaling. The type I receptor is a heterodimer, consisting of IL‐4Rα and the γ chain of the IL‐2 receptor, binding only to IL‐4. The type II receptor is a heterodimer formed by the IL‐4Rα and IL‐13Rα1, which binds to both IL‐4 and IL‐13, while IL‐13Rα2 is a decoy receptor that binds only to IL‐13 [35]. Upon receptor binding, the signal transducer and activator of transcription (STAT) 6 and STAT3 are activated, leading to the upregulation of genes, such as TSLP and IL‐24 in keratinocytes while genes for barrier‐related proteins, such as filaggrin (FLG), loricrin (LOR) and involucrin (IVL), are downregulated [35].

To mimic colonization rather than infection, we stimulated keratinocytes with heat‐killed bacteria, as the use of live bacteria would result in extensive keratinocyte death due to bacterial overgrowth in our experimental conditions. Our findings suggest that *S. aureus* may protect keratinocytes against HSV‐1 infection through several mechanisms. First, *S. aureus* induced the upregulation of antiviral pathways and innate immune responses, including the production of type I/III interferons and AMPs such as RNase 7 and hBD‐3. These molecules are known to restrict viral replication and spread, potentially contributing to reduced HSV‐1 susceptibility [33, 34, 36]. Additionally, *S. aureus* reduced the expression of key HSV‐1 entry receptors, including nectin‐1, HVEM, and syndecan‐1, which are critical for viral attachment, cell entry, and cell‐to‐cell spread [22, 23, 24, 37]. Together with the observed increase in HS3ST1 expression in the presence of bacteria, this suggests a potential shift towards alternative HSV‐1 entry mechanisms, possibly involving 3‐O‐sulfated heparin sulfate [31]. These combined effects likely hinder multiple stages of the HSV‐1 infection cycle. In contrast, *S. epidermidis* failed to elicit these protective effects, potentially due to differences in its ability to modulate the host immune response. Unlike *S. aureus* , *S. epidermidis* did not significantly upregulate antiviral genes nor alter the expression of HSV‐1 entry receptors in our model.

Toll‐like receptor 2 (TLR2) may play a role in explaining why *S. aureus* is protective against HSV‐1 infection. TLR2 is highly expressed on keratinocytes and is a key receptor for recognizing bacterial components like peptidoglycan, which is abundant in the cell wall of *S. aureus* [38]. Activation of TLR2 by *S. aureus* likely triggers downstream signaling pathways, including the induction of type I interferons, pro‐inflammatory cytokines such as IL‐6 and AMPs. However, it should be mentioned that *S. epidermidis* can induce AMPs via TLR2 as well [39].

While we observed a protective effect of *S. aureus* on HSV‐1 infection in keratinocytes in our study, similar antiviral responses have been reported following stimulation via other immune receptors. For instance, TLR3 activation by Poly (I:C) has been shown to restrict HSV infection in keratinocytes [40]. Therefore, keratinocytes stimulated via these innate immune pathways may reflect overlapping induction of antiviral genes, indicating that the observed effects may be explained by both canonical Pattern Recognition Receptor (PRR)‐mediated antiviral signaling and *S. aureus* ‐specific effects.

In line with our observations that *S. aureus* protects from subsequent HSV‐1 infections, Lin et al. showed the same effect of *S. aureus* lysates in human corneal epithelial cells, as well as in HSV‐1 infected murine corneas [41]. They found that the active antiviral components in *S. aureus* lysates appeared to influence the adhesion and replication processes of the viral particles, suggesting that *S. aureus* lysates may hinder the virus's ability to infect host cells but not necessarily directly impact the host cells themselves. However, the authors emphasized that the precise active components of *S. aureus* lysates remain unidentified, and while they speculated that proteins or glycoproteins could be involved, further research is required to pinpoint the specific agents responsible for the antiviral activity. In contrast, in vivo living *S. aureus* produces virulence factors such as toxins, proteases and superantigens, which actively disrupt the epidermal barrier and drive inflammation. Building on our observations based on the use of heat‐killed *S. aureus* and the matching effects of *S. aureus* lysates, future experiments using live bacteria co‐culture systems would further complement and strengthen these data. In light of these observations, an alternative mechanism likely modulates viral entry or replication. Exposure to *S. aureus* results in the downregulation of the HSV‐1 receptor nectin‐1 and, under Th2 cytokine conditions that simulate AD [42], also of herpesvirus entry mediator (HVEM). Viral entry requires the binding of viral glycoprotein D to one of its receptors, nectin‐1, which is expressed on keratinocytes; HVEM, a member of the tumor necrosis factor receptor family predominantly found on immune cells; or 3‐O‐sulfonated heparin sulfate [22, 43, 44, 45, 46, 47]. The downregulation of nectin‐1 and HVEM may thus impede viral entry.

Nectin‐1 is part of the nectin family and is an adherent junction molecule involved in cell–cell adhesions and via afadin connected to F‐actin of the cytoskeleton [15]. It can be hypothesized that a reduction in nectin‐1 expression disturbs the epithelial barrier and thereby contributes to AD.

Additionally, heat‐killed *S. aureus* also upregulates the expression of the antimicrobial peptides RNase 7 and human β‐defensin (hBD) 3, which possess antiviral properties [34, 36, 48, 49]. However, the efficacy of RNase 7 is compromised in AD patients due to the presence of increased levels of free DNA and RNase inhibitor [50]. Furthermore, patients with a history of EH were shown to be impaired in their ability to induce the expression of a variety of AMPs [51, 52].

While *S. aureus* is capable of producing a number of extracellular factors that contribute to the inflammation and barrier defect observed in AD, we hypothesize that the active compound of *S. aureus* that limits HSV‐1 infection might be isolated and harnessed as a therapeutic for patients at risk for EH [53]. The observed protective effect of IL‐4 and IL‐13 in this in vitro setting presents a paradox when contrasted with their role in vivo, where the Th2 milieu in AD is associated with increased susceptibility to HSV infections due to a type 2 polarized antiviral immune response [54]. This discrepancy may be explained by the absence of the complex environment found in vivo, where other factors such as barrier dysfunction, secondary infections, and chronic inflammation exacerbate viral susceptibility in AD, which cannot be displayed in a 2D model as used in this study, but would rather need a 3D setup. In contrast, in vitro keratinocytes are shielded from these compounding factors, allowing IL‐4/IL‐13 to induce certain protective pathways [34, 55]. Furthermore, in our study, we utilized a concentration of 40 ng/mL for both IL‐4 and IL‐13, which aligns with the range (10–50 ng/mL) used in previous studies to model Th2‐dominant inflammatory conditions [56, 57, 58]. Although these concentrations provide a robust and consistent inflammatory stimulus in vitro, they may not precisely reflect the levels encountered in vivo, where IL‐13 is higher expressed in AD lesional skin than IL‐4 [59, 60]. Theoretically, a different ratio of IL‐4 to IL‐13 in vivo could contribute to differences in susceptibility to infection compared to our in vitro model. However, this seems rather unlikely given that both receptors signal redundantly via STAT6. Future studies could incorporate advanced human‐based models, such as organotypic skin equivalents or ex vivo human skin. Furthermore, in vivo models could provide additional insights into systemic immune responses and immune‐epithelial‐microbiome interactions, as demonstrated by the murine model of EH described by Kawakami et al. [61]. In addition, recent studies have shown that non‐evasive topical application of *S. aureus* strains to the murine ear can efficiently induce immune responses and the formation of skin‐resident memory T cells [62].

In conclusion, this study reveals a reduced diversity of skin microbiota in AD patients with a history of EH, identifying *S. aureus* and *S. epidermidis* as prevalent species with differentially influence on HSV‐1 infection rates. Notably, *S. aureus* alone and in combination with Th2 cytokines reduced HSV‐1 infection in keratinocytes by modulating antiviral immune responses. *S. aureus* induced an “alarmed” state in undifferentiated, proliferating keratinocytes, resembling the basal layer of the epidermis. This state enabled the keratinocytes to reduce their own susceptibility to HSV‐1 infection independently of immune cells. *S. epidermidis* , however, was not able to induce this response. We speculate that this pre‐alarmed state, characterized by an interferon signature and a downregulation of herpesvirus entry factors, is a feature of keratinocytes exposed to the pathogenic *S. aureus* but not to commensals like *S. epidermidis* .

However, the pathogenicity of *S. aureus* may interfere with this response, particularly since ADEH^+^ patients have an even higher bias to *S. aureus* skin colonization. As the conditions in ADEH^+^ patients are more complex than reflected in our keratinocyte model, the protective, yet context‐dependent, immunomodulatory effects observed in this study may be masked by the impaired antiviral responses present in ADEH^+^ patients. Moreover, ADEH^+^ patients exhibit impaired AMP induction, suggesting they may fail to mount the protective AMP response observed in vitro despite strong *S. aureus* colonization. A patient‐stratified keratinocyte model using keratinocytes from ADEH^+^ patients to investigate *S. aureus* effects would allow us to reveal disease‐relevant heterogeneity in antiviral and innate immune responses, which was not feasible for the current study. Additionally, stratification of the genetic background would enable studies on genotype‐microbiome interactions. To investigate whether the altered skin microbiome in ADEH^+^ patients actually is a pre‐disposing state rather than resulting from the infection, additional longitudinal studies are required to investigate microbiome changes after EH episodes.

Future studies should investigate the intricate interplay between the host, HSV‐1, and *S. aureus* to better understand and potentially exploit *S. aureus* ' regulatory role in enhancing keratinocyte defenses against viral infections in the immunologically distinct environment of AD patients.

## Funding

The authors have nothing to report.

## Conflicts of Interest

The authors declare no conflicts of interest.

## Supporting information


**Figure S1:** Timespan between last eczema herpeticum episode and microbiome sampling in the ADEH^+^ cohort. Plot showing the years since the last occurrence of EH versus the total counts of patients.

**Figure S2:** Normalized cell viability of NHEKs after incubation with or without heat‐killed staphylococci. Cell Viability of NHEKs from different donors (*n* = 7) non‐stimulated (ns) or stimulated with different concentrations (OD 0.05–0.3) of (A) heat‐killed *S. epidermidis* (HKSE) and (B) heat‐killed *S. aureus* (HKSA) in the absence or presence of IL‐4/IL‐13 (40 ng/mL). Cell Viability was normalized to the mean of the control with or without IL‐4/IL‐13. Significances were tested for all conditions with a Repeated Measures ANOVA and Bonferroni post test and compared to the respective control. No significant differences were detectable (n.s.). Shown are the arithmetic means with whiskers. The Viability Assay was performed using the RealTime‐Glo MT Cell Viability Assay Kit (Promega) and luminescence was measured using a plate reader (FluoStar Optima, 37°C, 0.3 s settling time, 10 s measurement interval time).

**Figure S3:** 3D PCA plot and volcano plots of differentially expressed genes in HKSE‐ or HKSA‐treated or non‐stimulated keratinocytes. Transcriptomics analysis of keratinocytes stimulated by heat‐killed *S. aureus* (HKSA) and *S. epidermidis* (HKSE) in the absence of IL‐4/IL‐13. (A) 3D PCA analysis performed with the top *n* = 2000 genes selected by variance. (B) Volcano plot investigated differential expressed genes comparing HKSE stimulated keratinocytes to non‐stimulated keratinocytes and (C) comparing HKSA to HKSE stimulated keratinocytes. *n* = 3 independent NHEK donors/experiments.

**Figure S4:** Relative mRNA expression of interferon signaling pathway and proinflammatory cytokine genes, determined by RT‐qPCR. (A) IRF7 (B) IFNB1 (C) IFNL1 (D) IL1A and (E) IL1B of NHEKs stimulated with heat‐killed *S. aureus* (HKSA) or *S. epidermidis* (HKSE) or non‐stimulated (ns) in combination with or without IL‐4/IL‐13 (*n* = 6–7 independent experiments/NHEK donors). Mean with SD is shown. Friedman test with Dunn‘s multiple comparison test was performed for statistical analysis. * < 0.05, ** < 0.01.

**Figure S5:** Gene expression of interferon‐stimulated genes. (A) ISG15 (B) MX2 (C) EIF2AK2 (D) IFIT2 normalized counts of NHEKs stimulated with heat‐killed *S. aureus* (HKSA) or *S. epidermidis* (HKSE) or non‐stimulated (ns) in combination with or without IL‐4/IL‐13 (*n* = 3 independent experiments/NHEK donors). Mean with SD is shown. Friedman test with Dunn‘s multiple comparison test was performed for statistical analysis. * < 0.05.

**Figure S6:** KEGG Pathway of HSV‐1 infection in *S. aureus* stimulated keratinocytes without IL‐4/IL‐13. KEGG pathway “hsa05168” – Herpes simplex virus 1 infection depicted by the “pathview” function of the pathview package. Differential expressed genes comparing heat‐killed *S. aureus* (HKSA) stimulated keratinocytes with non‐stimulated keratinocytes without IL‐4/IL‐13 stimulation are depicted. Red: upregulated in HKSA stimulated cells. Green: downregulated in HKSA stimulated cells.

**Figure S7:** KEGG Pathway of HSV‐1 infection in *S. aureus* stimulated keratinocytes with IL‐4/IL‐13. KEGG pathway “hsa05168” ‐ Herpes simplex virus 1 infection depicted by the “pathview” function of the pathview package. Differential expressed genes comparing heat‐killed *S. aureus* (HKSA) stimulated keratinocytes with non‐stimulated keratinocytes with IL‐4/IL‐13 stimulation are depicted. Red: upregulated in HKSA stimulated cells. Green: downregulated in HKSA stimulated cells.

**Figure S8:** Cytokine production by NHEKs after stimulation with heat‐killed staphylococci in the presence or absence of IL‐4/IL‐13. (A) IL‐6, (B) IL‐8, (C) IL‐1α and (D) IL‐1β concentrations (pg/mL) in NHEK supernatants after stimulation with heat‐killed *S. epidermidis* (HKSE) or heat‐killed *S. aureus* (HKSA) in the presence or absence of IL‐4/IL‐13 (*n* = 7 independent experiments/NHEK donors). Data are shown as box plots with whiskers from minimum to maximum (bar, median). Significances were tested with a Friedman test and Dunn's multiple comparison post test and compared to the respective control, * < 0.05, ** < 0.01, *** < 0.001.

**Figure S9:** Relative mRNA expression of genes involved in HSV‐1 cell entry, determined by RT‐qPCR. (A) NECTIN1, (B) HVEM, (C) SDC1 and (D) HS3ST1 relative mRNA expression levels of NHEKs stimulated with heat‐killed *S. aureus* (HKSA) or *S. epidermidis* (HKSE) or non‐stimulated (ns) in combination with or without IL‐4/IL‐13 analyzed by RT‐qPCR (*n* = 7 independent experiments/NHEK donors). Mean with SD is shown. Friedman test with Dunn‘s multiple comparison test was performed for statistical analysis. * < 0.05, ** < 0.01.

**Data S1:** Methods.

## Data Availability

The data that support the findings of this study are available on request from the corresponding author. The data are not publicly available due to privacy or ethical restrictions.
