# Supplementary material for: Keratinocyte Priming by Staphylococcus aureus Reduces HSV‐1 Susceptibility
Source: Allergy. 2026 May 20;81(8):2843–57. doi: 10.1111/all.70392 (PMC13466154; doi:10.1111/all.70392)
Supplement: Supplementary file 1 [file ALL-81-2843-s006.docx]

**Supplementary methods**

## 16S microbiome analysis

Detailed methods regarding the swabs collected in the TREATgermany registry were described previously ^14^. Shortly, patients with moderate to severe AD were asked to avoid bathing, showering, or applying topical agents for 24 hours before the sampling visit. For the present analysis, the results from the swabs taken on the antecubital fossa were used.

Microbial DNA was isolated and the V1 and V2 variable regions of the 16S rRNA gene were amplified and sequenced. For sample processing, FastQC (version 0.11.9) was used for quality evaluation, and DADA2 (version 1.10) for inference of amplicon sequence variants (ASVs). For comparison, similarly sampled, extracted, and processed 16S rRNA sequence data from antecubital fossa skin swabs of 258 healthy participants of the PopGen cohort ^63^ were included in this study.

Statistical analyses were performed using R (version 4.2.3) ^15^ with an RStudio IDE (version 2023.03) ^16^ within a macOS Monterey environment. Microbiome data was analyzed using the phyloseq (version 1.46.0) ^17^, microbiome (version 1.24.0) ^64^, and vegan packages (version 2.6-8) ^18^. The Shannon index was calculated based on non-rarefied data.

## Keratinocyte cell culture

Human primary keratinocytes were prepared from foreskin as described before ^19^. Passages used varied from four to eight. Cells were cultured in keratinocyte growth medium 2 (Keratinocyte Basal Medium 2 plus supplements, PromoCell) without antibiotics at 37°C in a 5% CO_2_ atmosphere. Cells were passaged at around 80% confluence. For this, the medium was discarded and cells washed with DPBS (PAN-Biotech). EDTA (0.02%, PAN-Biotech) was added and incubated for approximately 5-10 min at 37°C. EDTA was discarded and trypsin/EDTA (0.05%/0.02%, PAN-Biotech) was added (~2 min, 37°C). Trypsin inhibitor (1 mg/mL, PAN-Biotech) was added, the flask was rinsed with DPBS, and the cells were collected by centrifugation at 20°C and 300 *g* for 4 min and washed with DPBS. Cells were then either split for further culture or plated for experiments.

## Vero cell culture

Vero CCL-81 cells were used in this work with passages from 23 to 29. Vero cells were cultured in MEM Eagle medium with Earle’s Balanced Salt Solution and L-glutamine (CytoGen GmbH) containing 7.5% FBS (Capricorn Scientific GmbH) and 1% Non-Essential Amino Acids (CytoGen GmbH) and incubated at 37°C in a 5% CO_2_ atmosphere. Cells were passaged at ~80-90% confluence as described for the keratinocyte cell culture, except that the trypsin inhibitor was omitted and Vero culture media was added instead.

## Bacterial culture and heat-killing

*S. aureus* (NCTC 8325) and *S. epidermidis* (DSM 20044, ATCC 14990) were plated on Columbia agar plates with 5% sheep blood (Becton Dickinson) and incubated at 37°C for 3 days until colonies were formed. Subcolonies were generated by picking one colony per strain, plated onto a new blood agar plate and incubated for about 24 h at 37°C. Then, 3-5 colonies of *S. aureus* and *S. epidermidis* subcolonies were picked from the blood agar plate and transferred to 8 mL of tryptic soy broth (TSB; Sigma-Aldrich) in a ventilated tube and incubated at 37°C in a shaker overnight. Overnight liquid cultures were diluted in TSB (1:10) and incubated on a shaker (37°C, 3 h). Bacterial solutions were centrifuged (20°C, 5000 *g*, 5 min) and washed with sterile 1 x DPBS.

Optical density (OD) was measured with a cell density meter (WPA Biowave CO8000, Biochrom). DPBS was used as reference. After another washing, the pellet was resuspended in keratinocyte basal medium (w/o supplements, w/ 0.06 mM CaCl_2_) with 100 μg/mL gentamicin and adjusted to the appropriate OD. An OD of 0.2 was used for HKSE and HKSA unless otherwise specified. Heat-killing was performed at 65°C for 1 h in a water bath. Successful heat-killing was confirmed by incubation (37°C, 5 days) of HKSE and HKSA streaked onto blood agar plates.

## Keratinocyte in vitro model

Keratinocytes were seeded into 96-well flat-bottom plates with a density of 1*10^4^ cells/well in 200 μL keratinocyte growth medium 2 (Keratinocyte Basal Medium 2 plus supplements, PromoCell) and incubated overnight. The next day, keratinocytes were stimulated with 40 ng/mL IL-4 and IL-13 (37°C, 24 h) or left untreated. The medium was aspirated from cultivated keratinocytes and 100 μL/well pre-heated (37°C) keratinocyte basal medium (w/o supplements, w/ 0.06 mM CaCl_2_) with 100 μg/mL gentamicin was added, followed by addition of 100 µL/well heat-killed *S. epidermidis* or *S. aureus*. Cells were incubated with heat-killed staphylococci overnight (37°C, 5% CO_2_).

## HSV-1 infection of keratinocytes

HSV1(17^+^)Lox-pMCMV-GFP ^65^ which expresses soluble GFP under control of an mouse cytomegalovirus immediate-early promoter, was diluted in CO_2_-independet medium w/ 0.1% BSA and kept on ice.

Supernatants were removed from keratinocytes and 100 μL/well chilled CO_2_-independent medium w/ 0.1% BSA was added to the cells for pre-cooling on ice for 10 min. Medium was removed and replaced with 50 μL/well inoculum with a viral dose of 1*10^6^ PFU/mL, followed by 2 h virus binding on ice on an oribital shaker (20 1/min). Keratinocytes were then washed three times with 100 μL/well CO_2_-ind. medium w/ 0.1% BSA (4°C), which was subsequently replaced with 100 μL/well keratinocyte basal medium (w/o supplements, w/ 0.06 mM CaCl_2_, with 100 μg/mL gentamicin) and were incubated at 37°C for 20 h.

HSV-1 supernatants were collected for later infection of Vero cells and stored at -80°C. Keratinocytes were fixed with 50 μL/well Roti-Histofix (4% formaldehyde solution, Carl Roth GmbH) and incubated for 20 min at room temperature. Cells were washed thrice with 100 μL/well DPBS and covered with 50 μL/well 300 nM DAPI in 0.1% TX-100 in DPBS and incubated for 10 min. Keratinocytes were washed twice with 100 μL/well DPBS and finally overlayed with 100 μL/well ddH_2_O. The plates were stored in the dark at 4°C until measurement of DAPI and GFP with an automated microscope (Cytation 3, BioTek Instruments Inc.).

## Vero cell infection

The supernatants collected from keratinocytes after 20 h of HSV-1 infection were used to infect indicator Vero cells to compare the amount of infectious HSV-1 released from the differentially treated keratinocytes. Vero cells (6000 cells/well in 50 µL Vero cell medium) were plated into 96-well plates and incubated overnight at 37°C. The medium was discarded and 50 μL of HSV-1 keratinocyte supernatant (1:2 in Vero cell medium) was added. After 20 h incubation at 37°C, the supernatant was removed and the Vero cells were fixed and stained as described above for further analysis.

## Microscopy and Image Analysis

Fixed HSV-1 infected keratinocytes and Vero cells were visualized via microscopy using the Cytation 3 cell imaging reader (BioTek Instruments Inc.) and further analyzed using the CellProfiler software ^66^. The Cytation reader was programmed to acquire twenty images per well (objective: 4x) with one channel set to detect DAPI (Excitation: 377 nm, Emission: 447 nm) and one set to detect GFP (Excitation: 469 nm, Emission: 525 nm) signals. Four images from the middle of each well were used for analysis to avoid background noise from the edges, and nuclei were identified via DAPI staining. The nuclei were then expanded to a defined size and the GFP intensities in this area were quantified.

## Cell viability assay

The RealTime-Glo™ MT Cell Viability Assay Kit (Promega) was performed after incubation with heat-killed *S. epidermidis* or *S. aureus* according to the manufacturer’s protocol. Briefly, all reagents and culture media were pre-warmed to 37°C. MT cell viability substrate and NanoLuc® enzyme were diluted in a ratio of 1:1000 in Keratinocyte Basal Medium (w/o supplements, w/ 0.06 mM CaCl_2_) with 100 μg/mL gentamicin. Supernatants were removed from the cells and 120 μL/well of viability reagent was added and incubated for 1 h at 37°C. Then, 100 μL of viability supernatant was transferred into a white 96-well plate to measure luminescence using a plate reader (FluoStar Optima, 37°C, 0.3 s settling time, 10 s measurement interval time).

## RNA isolation

RNA was isolated from keratinocytes infected with HKSE or HKSA using the ReliaPrep™ RNA Cell Miniprep System (Promega) according to the manufacturer’s protocol. Briefly, supernatants were removed from keratinocytes and cells were washed with ice-cold DPBS before adding 100 µL/well lysis buffer (BL buffer and 1-thioglycerol 1:100). The lysate was collected by repeated pipetting and transferred to a microcentrifuge tube. Isopropanol was added and vortexed for 5 s. The lysate was then transferred to a minicolumn, centrifuged and the liquid from the collection tube discarded. The minicolumn was washed with RNA Wash solution before DNase I was added to the minicolumn membrane and incubated for 15 min. The column was washed with Column Wash Solution and twice with RNA Wash Solution, then transferred to an elution tube and nuclease-free water was added to the minicolumn membrane to collect the purified RNA. RNA quantitiy was assessed using the NanoDrop 2000 (Thermo Scientific) and the 2100 Bioanalyzer (Agilent). RNA quality was controlled by determination of RNA integrity number (RIN) using the 2100 Bioanalyzer.

## Real-time quantitative PCR

cDNA was transcribed by reverse transcription using the RevertAid cDNA Synthesis Kit (Thermo Scientific) according to the manufacturer’s protocol. RT-qPCR analysis was performed using the LightCycler^®^ 480 (Roche) with the LightCycler^®^ 480 SYBR Green Kit (Roche) or QuantiTect SYBR Green PCR Kit (Qiagen) and the QuantiTect primers (Qiagen) for RNase7 (QT00239463), DEFB103A (QT01529535), RPS20 (QT00003290), IRF7 (QT00210595), IFNB1 (QT00203763), IFNL1 (QT01033564), IL1A (QT00001127), IL1B (QT00021385), PVRL1/NECTIN1(QT00054845), TNFRSF14/HVEM (QT00082432), SDC1 (QT00037128) and HS3ST1 (QT00071309) according to the manufacturer’s protocol but only using half of the recommended amounts (5 µL SybrGreen, 3 µL H_2_O, 1 µL Primer, 1µL cDNA). The amount of target mRNA relative to the amount of the reference RPS20 mRNA within the same sample was calculated using the LightCycler^®^ 480 Software (Roche) and normalized to the non-stimulated control of one donor.

## Library generation, quality control, and quantification

160 ng of total RNA per sample was used as input for mRNA enrichment procedure with ‘NEBNext® Poly(A) mRNA Magnetic Isolation Module’ (E7490L; New England Biolabs) followed by stranded cDNA library generation using ‘NEBNext® Ultra II Directional RNA Library Prep Kit for Illumina’ (E7760L; New England Biolabs). All steps were performed as recommended in the E7760 user manual (Version 1.0_02-2017; NEB) except that all reactions were downscaled to 2/3 of the original volumes. cDNA libraries were barcoded by dual indexing approach, using ‘NEBNext Multiplex Oligos for Illumina – 96 Unique Dual Index Primer Pairs’ (6440S; New England Biolabs). All generated cDNA libraries were amplified with 9 cycles of final PCR. One additional purification step was added at the end of the standard procedure, using 1.2x ‘Agencourt® AMPure® XP Beads’ (#A63881; Beckman Coulter, Inc.). The fragment length distribution of individual libraries was monitored using ‘Bioanalyzer High Sensitivity DNA Assay’ (5067-4626; Agilent Technologies). Quantification of the libraries was performed using the ‘Qubit® dsDNA HS Assay Kit’ (Q32854; ThermoFisher Scientific).

## Library denaturation and Sequencing run

Equal molar amounts of individually barcoded libraries were pooled for a common sequencing run, with each library analyzed representing around 5.6% of the total flowcell / run capacity. The library pool was denatured with NaOH and was finally diluted to 1.8 pM according to the Denature and Dilute Libraries Guide (Document # 15048776 v02; Illumina). 1.3 mL of the denatured pool was loaded onto an Illumina NextSeq 550 sequencer using a High Output Flowcell (400M cluster) for single reads (20024906; Illumina). Sequencing was performed with the following settings: Sequence reads 1 and 2 with 38 bases each; Index reads 1 and 2 with 8 bases each.

## BCL to FASTQ conversion

BCL files were converted to FASTQ files using bcl2fastq Conversion Software version v2.20.0.422 (Illumina).

## Raw data processing and quality control

Raw data processing was conducted using nfcore/rnaseq (version 3.9), a bioinformatics best-practice analysis pipeline used for RNA sequencing data at the National Genomics Infrastructure at SciLifeLab Stockholm, Sweden. The pipeline uses Nextflow, a bioinformatics workflow tool ^67^. It pre-processes raw data from FastQ inputs, aligns the reads, and performs extensive quality control on the results. The genome reference and annotation data were obtained from GENCODE.org (Homo sapiens: GRCh38.p13; release 39).

## Normalization and differential expression analysis

Normalization and differential expression analysis were performed on the internal Galaxy (version 20.05) instance of the RCU Genomics, Hannover Medical School, Germany with DESeq2 (Galaxy Tool Version 2.11.40.6) ^68^ with default settings except for “Output normalized counts table”, which was set to “Yes” and all additional filters were disabled (“Turn off outliers replacement”, “Turn off outliers filtering”, and “Turn off independent filtering” set “Yes”). The different conditions were selected as the primary factor, whereas the batch was used as the secondary factor in DESeq2 analyses (two factor design). Analysis was performed with multiple levels of the primary factor (all different stimulation conditions) comparing all levels against each other.

## RNA sequencing analysis

RNA-seq data analysis was performed using R (sse above) with packages from Bioconductor (www.bioconductor.org). Count data were imported from a pre-processed Salmon-pipeline output. Differential expression analysis was conducted using the DESeq2 package. A DESeqDataSet was created from the count data and metadata using the design formula ~group. Genes with fewer than 10 reads across all samples were filtered out. Fold-change shrinkage was applied using the apeglm method, and differentially expressed genes (DEGs) with a log2 fold-change (LFC) > 2 and an adjusted p-value (FDR) < 0.05 were identified.

Adjusted p-values were calculated using the Benjamini–Hochberg procedure. DEGs were annotated with gene symbols using the AnnotationDbi package (version 1.64.1) ^69^.

A variance stabilizing transformation (VST) was applied to the count data using vst from DESeq2. PCA was performed on the top 2000 most variable genes, and clustering was visualized with the default plotPCA function. For more detailed visualization, prcomp was used, and the top three principal components were plotted in 3D using the scatterplot3d package. The clusterProfiler package (version 4.10.1) ^70^ was used for pathway analyses.

## Statistical analysis

For statistical analysis the software Graph Pad Prism^®^ 8.4.3 (GraphPad Software Inc) was used. Data were analyzed for normal distribution using the Shapiro-Wilk-test. Normally distributed data were analyzed with Repeated Measures ANOVA with Dunnett’s multiple comparison test and data with a non-Gaussian distribution were analyzed with Friedman test for paired data and uncorrected Dunn’s multiple comparison test or Kruskal-Wallis test for unpaired data. *P* values ≤ 0.05 were considered as significant. **p* < 0.05, ***p* < 0.01, ****p* < 0.001, *****p* < 0.0001.

## References

63. L. Moitinho-Silva, N. Boraczynski, H. Emmert, et al., “Host Traits, Lifestyle and Environment Are Associated With Human Skin Bacteria,” *British Journal of Dermatology* 185 (2021): 573–584, https://doi.org/10.1111/bjd.20072.
64. “Bioconductor—Microbiome (Development Version) [Internet],” (2025), https://bioconductor.org/packages/devel/bioc/html/microbiome.html.
65. B. Snijder, R. Sacher, P. Rämö, et al., “Single-Cell Analysis of Population Context Advances RNAi Screening at Multiple Levels,” *Molecular Systems Biology* 8 (2012): 579, https://doi.org/10.1038/msb.2012.9.
66. A. E. Carpenter, T. R. Jones, M. R. Lamprecht, et al., “CellProfiler: Image Analysis Software for Identifying and Quantifying Cell Phenotypes,” *Genome Biology* 7 (2006): R100, https://doi.org/10.1186/gb-2006-7-10-r100.
67. P. Di Tommaso, M. Chatzou, E. W. Floden, P. P. Barja, E. Palumbo, and C. Notredame, “Nextflow Enables Reproducible Computational Workflows,” *Nature Biotechnology* 35 (2017): 316–319, https://doi.org/10.1038/nbt.3820.
68. M. I. Love, W. Huber, and S. Anders, “Moderated Estimation of Fold Change and Dispersion for RNA-Seq Data With DESeq2,” *Genome Biology* 15 (2014): 1–21, https://doi.org/10.1186/S13059-014-0550-8.
69. “Bioconductor—AnnotationDbi [Internet],” (2025), https://bioconductor.org/packages/release/bioc/html/AnnotationDbi.html.
70. T. Wu, E. Hu, S. Xu, et al., “Clusterprofiler 4.0: A Universal Enrichment Tool for Interpreting Omics Data,” *Innovation (Cambridge, Mass)* 2 (2021): 100141, https://doi.org/10.1016/j.xinn.2021.100141.
